# Rumen-Reticular Temperature During Estrus and Ovulation Using Automated Activity Monitors in Dairy Cows

**DOI:** 10.3389/fvets.2020.597512

**Published:** 2020-12-16

**Authors:** Tracy A. Burnett, Manveen Kaur, Liam Polsky, Ronaldo L. A. Cerri

**Affiliations:** Applied Animal Biology, Faculty of Land and Food Systems, University of British Columbia, Vancouver, BC, Canada

**Keywords:** ovulation, automated technology, dairy cattle, estrous expression, rumen-reticular temperature

## Abstract

This study aimed to determine changes in rumen-reticular temperature (RRT) at estrus or ovulation and determine if these changes are associated with the intensity of estrous expression. Cows were equipped with an automated activity monitor (AAM) and a rumen-reticular bolus thermometer. A total of 190 estrus episodes were used where physical activity data was recorded using the AAM and ovulation was determined via ultrasonography of the ovaries at alert and twice daily, for a maximum of 60 h. Estrous expression was assessed using the maximum activity and duration in which activity remained above the AAM threshold; both characteristics were categorized using the median. Temperature data was collected for the duration of estrus, as well as for the interval of time where ovulation was determined to occur. Three measures of temperature were calculated: (1) positive area under the curve (AUC), (2) maximum positive temperature change (PTC), (3) maximum negative temperature change (NTC) at estrus (AUC_E_/ PTC_E_/ NTC_E_) and around ovulation (AUC_O_/PTC_O_/ NTC_O_). Both AUC and PTC were greater during estrus than around ovulation (2.7 ± 0.2 and 1.1 ± 0.3°C^2^ for AUC_E_ and AUC_O_; 0.55 ± 0.03 and 0.26 ± 0.04°C for PTC_E_ and PTC_O_, respectively). In contrast, NTC was lower around ovulation than estrus (−0.28 ± 0.05 and −0.60 ± 0.06°C for NTC_E_ and NTC_O_). Cows with greater estrous expression had greater AUC and PTC during estrus than around ovulation, but cows with lesser estrous expression had similar AUC and PTC. Increases in AUC [High THI (Temperature Humidity Index): High activity: 4.7 ± 0.5, Low activity: 1.5 ± 0.4; Low THI: High activity: 3.1 ± 0.2, Low activity: 1.4 ± 0.2 °C^2^] and PTC (High THI: High activity: 0.79 ± 0.08, Low activity: 0.36 ± 0.07; Low THI: High activity: 0.60 ± 0.04, Low activity: 0.47 ± 0.04°C) associated with estrous expression were found to be greater on days with higher THI. Alerts created using standard deviations from the mean were unable to detect estrus or ovulation with acceptable precision. Further research is required to determine how changes in RRT can be used successfully to predict estrus and ovulation.

## Introduction

New technologies have been developed to improve dairy cattle health and reproductive efficiency on dairy farms. The development of hormonal protocols such as the Ovsynch have greatly improved reproductive efficiency in lactating dairy cows by increasing insemination rates, but a growing body of research indicates that insemination programs based on the detection of estrus are equally effective at obtaining acceptable conception rates and days to conception (1–3). The monitoring of core body temperature to assess febrile states has been widely used in human and veterinary sciences. The recent development of rumen-reticular thermometer technologies that monitor core body temperature provide continuous data collection and analysis in real time. The ability to automatically measure continuous body temperature expands the applicability for health monitoring, and has the potential to also be used as a predictor of estrus and ovulation. Vaginal temperature increases near the onset of estrus and decreases near ovulation (4–6). The recent surge in interest and use of automated technologies for estrus detection requires that these technologies be validated in terms of their performance.

Methods of determining ovulation are limited by the need for either blood or milk hormone analyses at multiple time points or transrectal ultrasonography of the ovaries; neither procedure is able to detect the precise time of ovulation. The measurement of the LH surge, which precedes ovulation by ~26 h (7) with 0.5 h deviations (7), is the key event preceding ovulation. However, to be able to measure the LH surge, blood samples need to be taken at short intervals over several hours, thus limiting its practical use on farms. Although the timing of ovulation can be estimated using ultrasonography, this methodology also requires a substantial time investment (e.g., scanning once every 3 h). In contrast, automated activity monitors (AAM) can accurately detect behavioral changes that are associated with estrus, such as increased number of steps (8), and reduced rumination times (9), changes in feed intake (10), and lying time (11). However, variation in the time to ovulation relative to the onset of estrus remains a critical factor for a successful pregnancy.

The goals of this study were to (1) determine if changes in rumen-reticular temperature during estrus or ovulation exist and, (2) determine if these changes are impacted by the intensity of estrous expression. Our hypothesis was that increased physical activity during estrus detected using an automated activity monitor would be associated with increased rumen-reticular temperature, but a decrease in temperature would be captured near ovulation by the rumen-reticular temperature logger.

## Materials and Methods

This experiment was conducted at The University of British Columbia's Dairy Education and Research Center, Agassiz, BC. All procedures were approved by the University of British Columbia's Animal Care Committee (protocols # A15-0089 and #A10-0290).

### Animals and Housing

The cows used in the present study were a subset of animals initially described in a companion study (12). To be included in the present study cows had to be 30 DIM, had an estrus alert on the AAM, and be equipped with a rumen-reticular temperature bolus. During the experimental period, a total of 225 events were alerted as in estrus from 102 different animals. A total of 190 true episodes of estrus arising from 97 lactating Holstein cows, each having on average 1.9 ± 1.1 (mean ± SD) estrus episodes were used for the analysis of this study. Cows had a mean (± SD) DIM of 98 ± 57, parity of 2.4 ± 1.6, BCS of 2.78 ± 0.20, GS of 2.3 ± 0.70 and a 305-d mature-equivalent yield of 12,600 ± 1,700 kg of milk at each estrus event; 31.6% of the estrus events used were from primiparous cows. Animals were housed in a naturally ventilated wooden-framed barn with a free-stall design, equipped with deep sand-bedded stalls and milked twice daily at 05:00 and 15:00 h in a parallel milking parlor. Fresh TMR was delivered twice daily at ~07:00 and 16:00 h. The TMR was formulated following the NRC guidelines (13) to meet or exceed the requirements of a 620 kg Holstein cow producing 40 kg/d of 3.5% fat corrected milk; all animals had *ad libitum* access to both TMR and water. All cows had their body condition and gait scored at the time of enrolment into the study. Body condition was scored on a 5-point scale from thin (1) to obese (5) as outlined by Edmonson et al. (14). Cows were later classified as thin (<2.75), average (= 2.75), or moderate (> 2.75). Gait score (GS) was determined on a 5-point scale from normal (1) to severely lame (5) as outlined by Flower and Weary (15). Animals were later classified as sound (≤ 2) and lame (> 2). Health and production information was collected by the dairy herd personnel with the assistance of the herd veterinarian, and confirmed and recorded by the project leader using the on-farm Dairy Comp 305 software (Valley Agricultural Software, Tulare, CA).

### Study Design

In this observational cohort study, animals were continuously monitored by a collar-mounted automated activity monitor (Heatime®, H-Tags, SCR Engineers, Netanya, Israel) that was fitted on the upper left side of the neck beginning 10 d after parturition; activity was recorded in 2 h blocks and checked twice daily at milking times to determine when cows had crossed the alert threshold. Cows were enrolled onto the study when the monitor identified them as having crossed the alert threshold, set at 35 index; an index activity of 35 equates to approximately a 6-SD change in activity compared with a baseline set by the AAM system. Peak activity (maximum activity during an estrus episode) and duration (amount of time the animal spent with activity above the threshold) were used to describe the expression of estrus [see also Madureira et al. (8)]. Animals confirmed as in estrus were then monitored for ovulation using ultrasonography immediately following each milking (twice daily) for a maximum of 6 examinations (~60 h after the first ultrasound).

Weighted rumen-reticular thermometer boluses (TempTrack®, DVM Systems, Greeley, CO) were administered orally into the digestive tract of the cows using a specialized gun at 35 ± 7 DIM. Associated software allowed for hourly temperature monitoring. Environmental temperature and humidity were recorded hourly using an onsite weather station (Agriculture and Agri-Food Canada, Agassiz, BC). The Temperature Humidity Index (THI) was calculated following Mader et al. (16): THI = [0.8 * Temperature (°C)] + [(Humidity/100)*(Temperature −14.3)] + 46.4. Maximum THI was determined for the early morning (00:00 to 07:59 h), morning (08:00 to 15:59 h), and evening (16:00 to 23:59 h) periods; the THI at the time of onset of estrus and ovulation were determined based on these periods.

### Determination of Estrus, Ovulation, and Ovulation Times

Cows had their ovaries examined by ultrasound (Ibex Pro; E.I. Medical Imaging, Loveland, CO) using a 7.5 MHz linear-array rectal probe at enrolment, and then twice daily at milking times, at ~08:00 and 17:00 h, until a maximum of 6 per rectum examinations had occurred (~60 h after the first palpation); the average interval between scans was 11.8 ± 4 h. The presence and diameter of the 3 largest follicles and corpus lutea were measured and recorded. Cows were classified as in estrus if they had at least one dominant pre-ovulatory follicle >15 mm and an absence of a large corpus luteum (>20 mm) at the time of the estrus alert. Ovulation was determined by the disappearance of the dominant pre-ovulatory follicle; the time of ovulation was determined as the median time between the two ultrasound examinations where the pre-ovulatory follicle disappeared. Ovulation intervals were calculated as the time from when an alert on the AAM occurred until the time of ovulation.

All animals with estrus alerts had their ovaries scanned using ultrasonography 7 days after their last scan for the presence of a new corpus luteum which was used for confirmation ovulation. A new corpus luteum was used to confirm ovulation for true estrus alerts that were followed by ovulation. For estrus alerts without ovulation by 6 exams, the presence of a new corpus luteum was used to determine late ovulations and the absence of a new corpus luteum was indicative of failed ovulations.

### Rumen-Reticular Temperature Data

Changes in rumen-reticular temperature at estrus were determined for the entire duration of estrus, using the onset and end of estrus as indicated by the AAM. To determine changes in rumen-reticular temperature around ovulation, temperature data were collected from the period between the ultrasound examination that confirmed ovulation and the preceding exam. The positive area under the curve (AUC), maximum temperature change (PTC; i.e., positive amplitude), minimum temperature change (NTC; i.e., negative amplitude) were calculated at estrus (AUC_E_/PTC_E_/NTC_E_) and around ovulation (AUC_O_/PTC_O_/NTC_O_) relative to baseline. Baseline values were calculated hourly using the previous 5 d of hourly temperature readings after they had been corrected for bouts of water intake using a proprietary manufacture's algorithm (TempTrack®, DVM Systems, Greeley, CO). Measures of AUC were used as a method of measuring the intensity and length of time each animal spent above their baseline temperature during estrus and ovulation. The AUC was calculated using the trapezoidal rule, where only positive areas were considered, following the following formula:

$$\sum_{i=1}^{n}(x_{i+1}-\;x_{i})\;*\;(y_{i+1}+y_{i})/2$$

where *x* is the observed time stamp of the temperature reading to the nearest second, *y* is the observed rumen-reticular temperature relative to baseline in Celsius (°C), beginning at the first temperature reading of the sampling time (*i*=1) until the last (*n*th) observation.

### Estrus and Ovulation Alerts Using Changes in Rumen-Reticular Temperature

**Figure 1:**
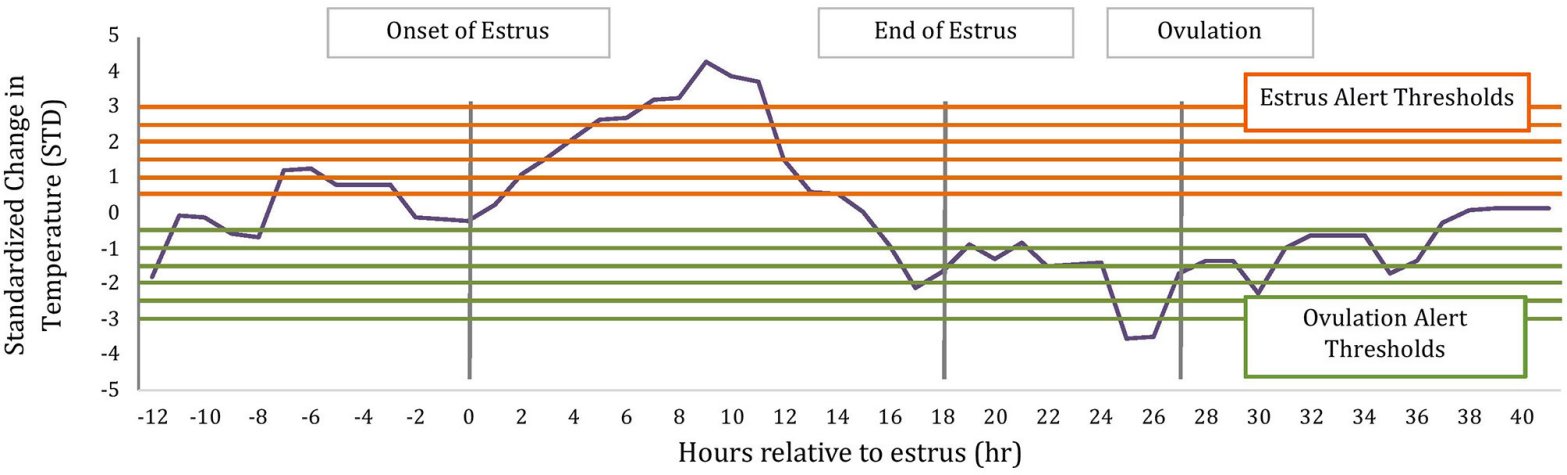
Schematic of estrus and ovulations alerts based on standardized change in rumen-reticular temperature relative to baseline. Horizontal lines represent alerting thresholds of 0.5, 1, 1.5, 2, 2.5, and 3 STD and −0.5, −1, −1.5, −2, −2.5, −3 STD for estrus and ovulation temperature alerts, respectively. Temperature alerts were determined on all data points between 12 h before the AAM estrus alert until 12 h after ovulation.

To determine if temperature changes around estrus could be potentially used within an alerting system for ovulation timing, we calculated the magnitude of change in temperature relative to a rolling baseline value using the following formula: (Temperature – Baseline Temperature)/Baseline Standard Deviation. Similar to above, the baseline values and standard deviations were calculated hourly using the previous 5 d of hourly temperature readings after they had been corrected for bouts of water intake using a proprietary manufacture's algorithm (TempTrack®, DVM Systems, Greeley, CO); water intake was corrected based off previous research (17). Temperature data for alerts were collected from 12 h before the AAM alert until 12 h after ovulation. Ovulation has previously been associated with a drop in temperature (18) thus alerts were then categorized as estrus or ovulation alerts based on the direction of change, where estrus alerts were of positive change and ovulation of negative change as depicted in Figure 1.

### Statistical Analysis

All statistical analyses were carried out with SAS Studio (version 5.1; SAS Inst. Inc., Cary, NC) with estrus episode as the experimental unit. The data were examined for missing data points and events missing >25% of the hourly data points, either at estrus or around ovulation, were excluded from the analysis. Estrus events that were confirmed as false and those with failed ovulations were also removed from the analysis. During the experimental period, a total of 225 estrus events from 102 different cows were alerted using automated activity monitors from cows with functioning rumen-reticular boluses, where 12.9% (*n* = 29) were removed due to being false alerts, and another 2.6% (*n* = 6) were removed due to having >25% missing temperature data points at both estrus and ovulation. Before analyses, data were checked for normality using the UNIVARIATE procedure and probability distribution plots; no outliers were found within dependant variables.

Peak activity was categorized using the median of 80 index into high (80–100 index) and low (35–79 index) intensity of events. Duration of estrus was also categorized by the median, 12 h, into short and long estrus events. THI was determined for the respective sampling period (onset of estrus and ovulation) and categorized as high and low using a THI index of 72. Cows with more than one lactation were classified as multiparous and those with only one lactation were considered primiparous.

The AUC, PTC, and NTC were used as dependent variables and were tested for the effects of sampling time (at estrus vs. around ovulation), estrous expression (peak and duration), THI, parity, BCS, GS, and stage of lactation using ANOVA with sampling time as a repeated measure and estrus event nested in cow as a random effect using the MIXED procedure. Due to collinearity, peak activity and duration were modeled separately (*r* = 0.78; *P* < 0.001). All multivariable models were constructed using the variables as described above and manual backward stepwise elimination was used, where variables were retained with *P* ≤ 0.15. Interaction were tested between all variables selected in the final model and kept if *P* < 0.05. Descriptions of final linear mixed regression models have been summarized in Table 1.

**Table 1:** Description of final linear mixed regression models where estrus event was used as a random intercept and sampling time was used as a repeated measure for the outcome variables of temperature change: area under the curve, maximum temperature change, and minimum temperature change.

**Outcome**	**Independent variable**	***P*-value**
Positive area under the curve (°C^2^)	Sampling time^1^	<0.001
	Peak activity^2^ OR activity duration^3^	<0.01
	Sampling time*^4^ peak activity OR activity duration (See Figures 3, 4)	<0.001
	Sampling time* peak activity OR activity duration* THI^5^ (See Figure 5)	<0.01
Maximum positive rumen-reticular temperature change (°C)	Sampling time	<0.001
	Peak activity OR activity duration	
	THI	0.03
	Milk production^6^	0.04
	Sampling time* peak activity OR activity duration (See Figures 3, 4)	<0.01
	Sampling time* peak activity OR activity duration* THI (See Figure 6)	<0.01
Maximum negative rumen-reticular temperature change (°C)	Sampling time	<0.001
	Parity^7^	0.08
	OR	
	Sampling time	<0.001
	Parity	0.04
	Sampling time* activity duration (See Figure 4)	<0.01

*Temperature changes were calculated over the duration of estrus, as measured using an automated activity monitor (AAM), and surrounding the time of ovulation using ovarian data. Estrous expression parameters, peak activity and duration, were modeled separately due to collinearity*.

1 *Sampling time refers to the period which temperature change data was collected, either at estrus, or around ovulation*.

2 *Peak activity refers to the maximum change in activity measured by the AAM during estrous. Peak activity was categorized using the median (80 index) into high and low peak activity*.

3 *Duration refers to the number of hours the cow surpassed the alert threshold on the AAM. Duration was categorized using the median (12 h) into long and short episode durations*.

4 Asterix (^*^) denotes an interaction.

5 *Maximum temperature humidity index (THI) was determined for the morning (00:00 to 07:59 h), afternoon (08:00 to 15:59 h), and night (16:00 to 23:59 h) periods and subsequently matched to the periods corresponding to the onset of estrus or ovulation periods of each cow. THI was categorized using the THI index of 72 into high or low THI*.

6 *Milk production was measured as the 305-d mature-equivalent yield in kg*.

7 *Parity was defined as primiparous (first lactation) or multiparous (2^nd^ lactation or greater)*.

Estrus and ovulation alerts were determined for positive and negative changes in rumen-reticular temperature relative to baseline, respectively. An alert was considered as two temperature readings in a row (i.e., 2 h in a row) that exceeded the following thresholds: 0.5 STD, 1 STD, 1.5 STD, 2 STD, 2.5 STD, or 3 STD. Time intervals were calculated between the two types of temperature alerts and the AAM alert for estrus and the timing of ovulation based off of ovarian dynamics, for each of the set thresholds. When more than one temperature alert was created for a given threshold within a cow, the first alert issued was used to create time intervals.

## Results

### Rumen-Reticular Temperature

**Figure 2:**
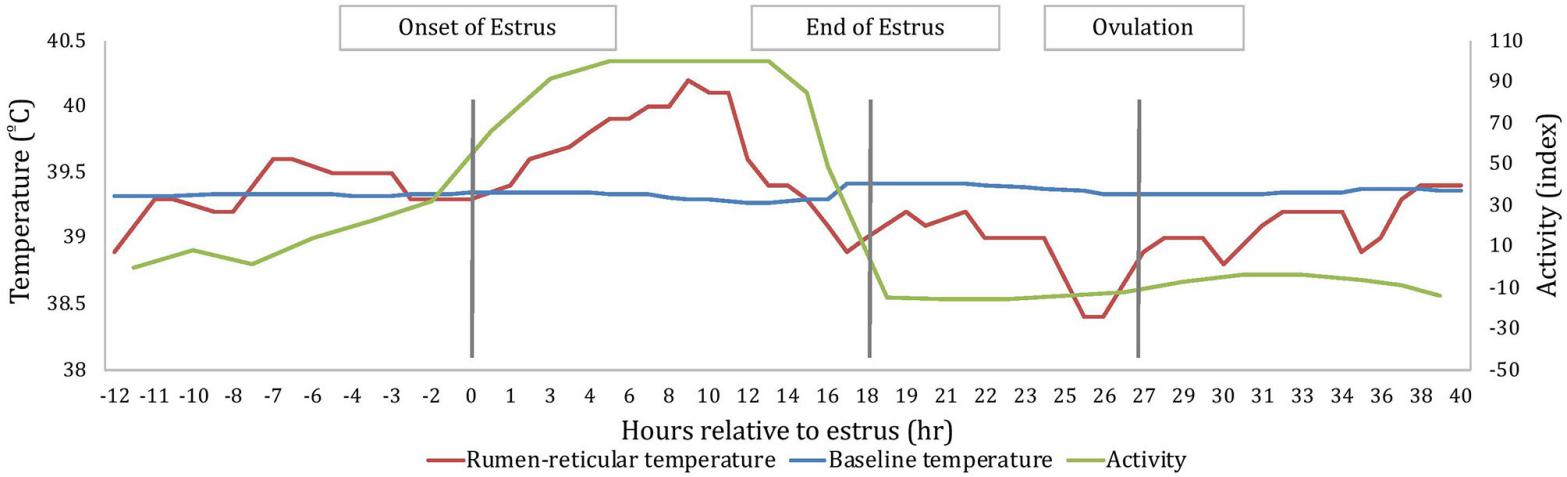
An example depiction, based on one cow, of the variation in rumen-reticular temperature, baseline rumen-reticular temperature, and activity change from the automated activity monitor per hour relative to estrus. Onset and end of estrus is based on the automated activity monitor, while the timing of ovulation is based on ovarian dynamics.

All measures of temperature change (AUC, PTC, and NTC) were affected by sampling time; where AUC and PTC were greater at estrus than around ovulation (2.7 ± 0.2 and 1.1 ± 0.3°C^2^ for AUC_E_ and AUC_O_, respectively; *P* < 0.001; 0.55 ± 0.03 and 0.26 ± 0.04°C for PTC_E_ and PTC_O_, respectively; *P* < 0.001) and NTC was more negative at ovulation than at estrus (−0.28 ± 0.05 and −0.60 ± 0.06 °C for NTC_E_ and NTC_O_, respectively; *P* < 0.001). An example of changes of rumen-reticular temperature relative to estrus and ovulation of one cow has been depicted in Figure 2.

**Figure 3:**
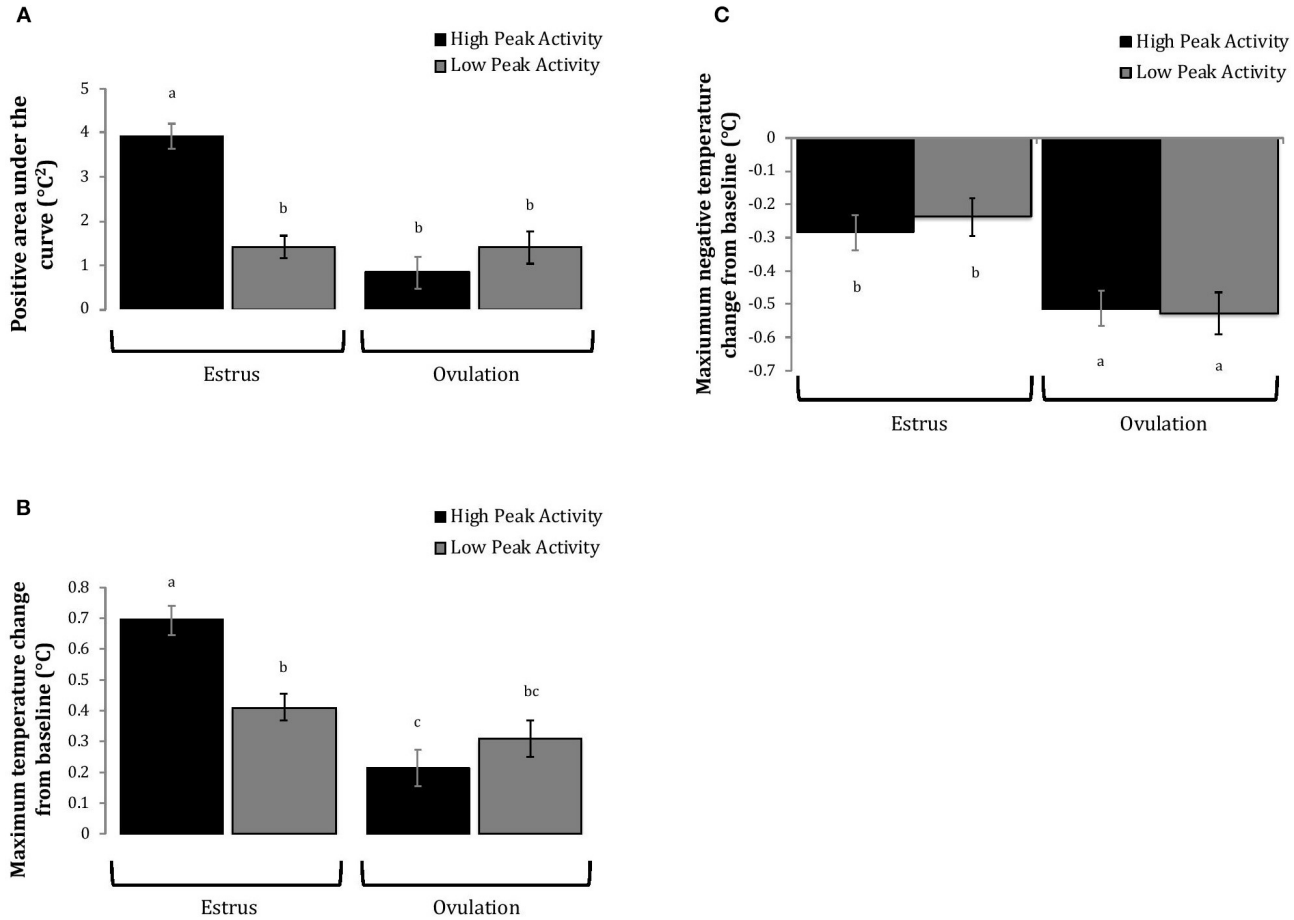
Changes in rumen-reticular temperature relative to baseline (least squared means + SE) during estrus and near ovulation in Holstein dairy cows expressing a high or low intensity of estrous expression, obtained from a mixed linear regression model as described in Table 1. The following three temperature changes are depicted: positive area under the curve [**(A)**; *P* < 0.001], maximum change [**(B)**; *P* < 0.01], and maximum negative change [**(C)**; *P* = 0.73). Superscripts of letters a-c denote significant differences (*P* < 0.05) between all groups of each panel. High peak activity: estrous expression greater than the median of 80 index on the AAM. Low peak activity: estrous expression less than the median.

**Figure 4:**
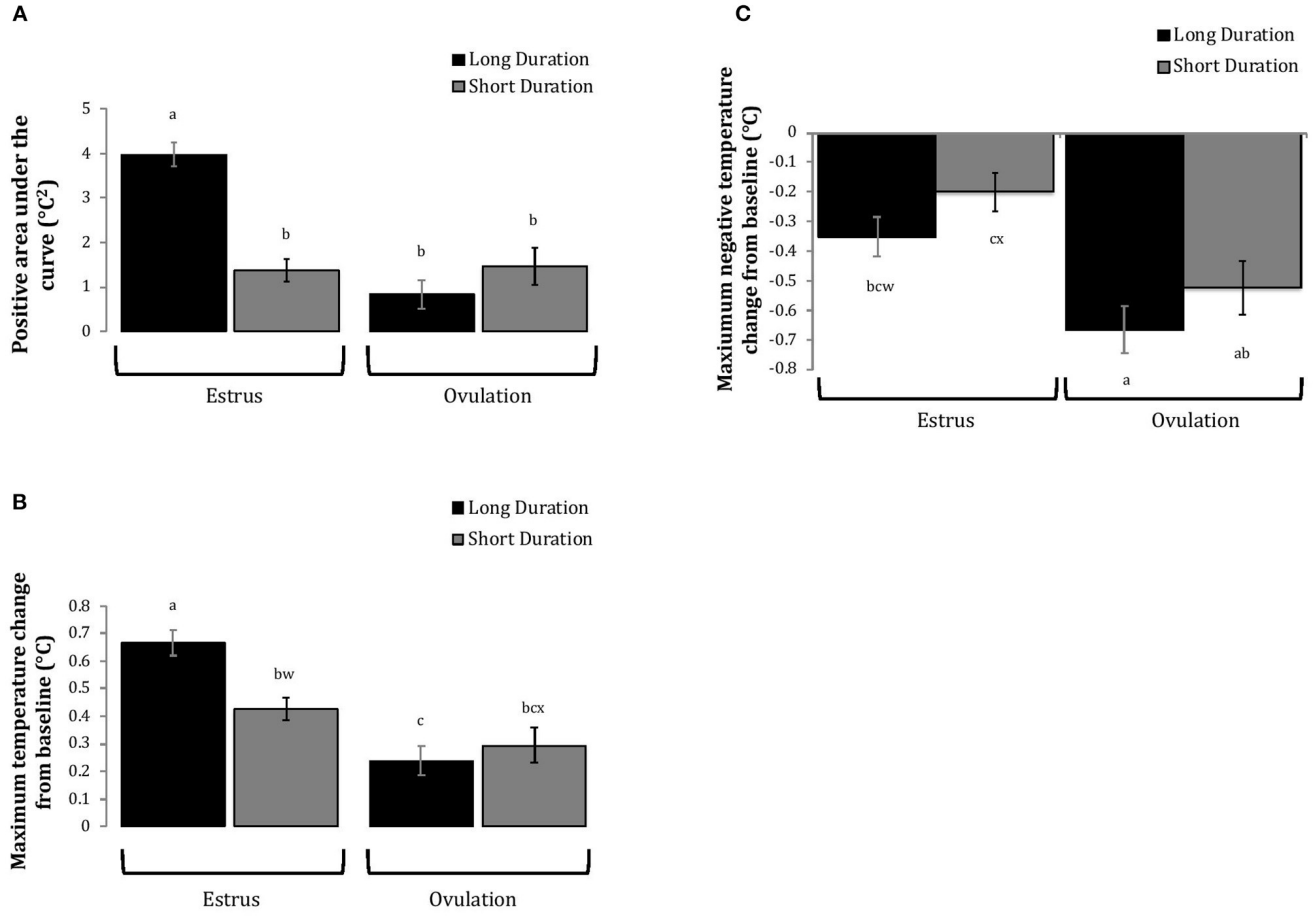
Changes in rumen-reticular temperature relative to baseline (least squared means± SE) during estrus and near ovulation in Holstein dairy cows expressing a long or short durations of estrous expression, obtained from a mixed linear regression model as described in Table 1. The following three temperature changes are depicted: positive area under the curve [**(A)**; *P* < 0.001], maximum change [**(B)**; *P* < 0.01], and maximum negative change [**(C)**; *P* < 0.01]. Superscripts of letters a-c denote significant differences (*P* < 0.05), while letters w-x denote tendencies (0.05 ≥ *P* < 0.10), between all groups of each panel. Low peak activity: estrous expression less than the median. Long duration: estrous expression lasting longer than the median of 12 h.

**Figure 5:**
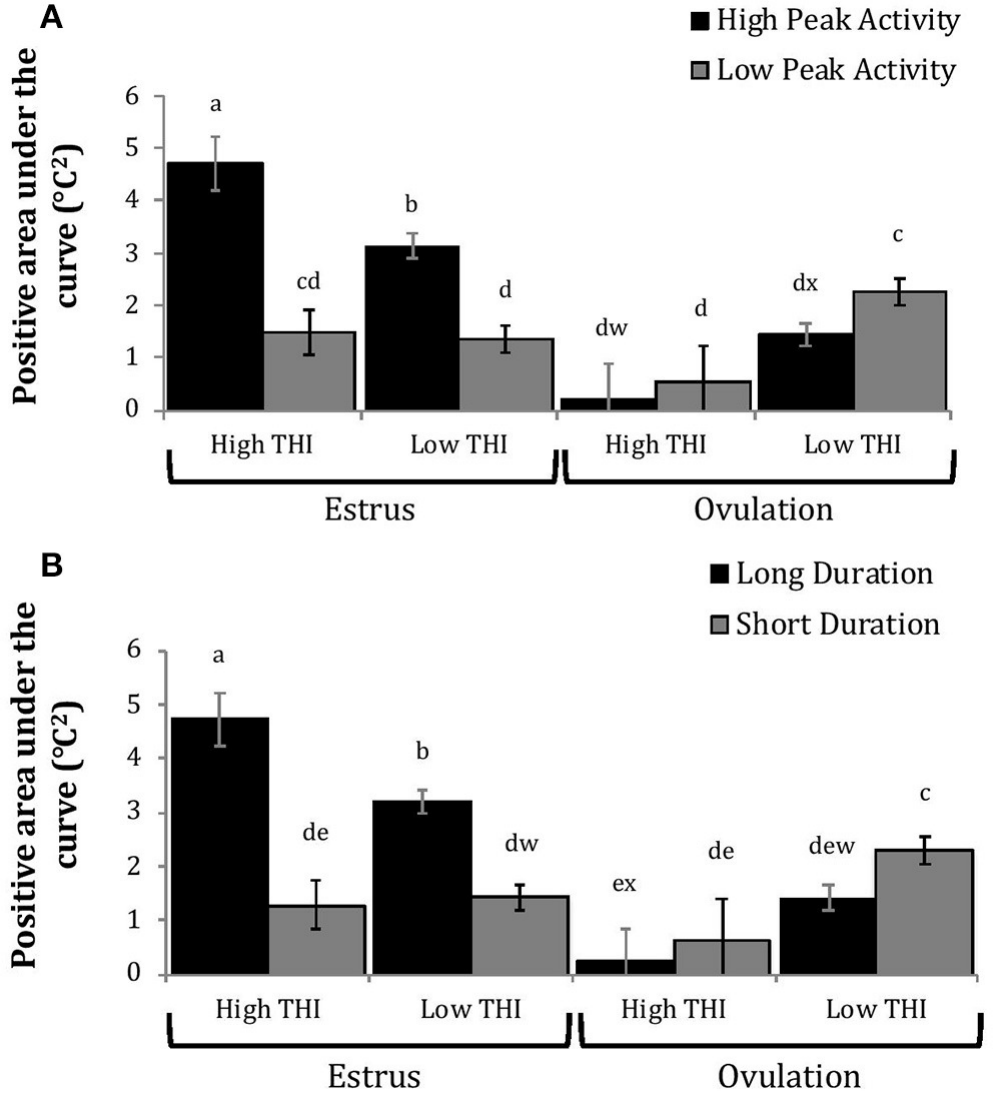
Positive area under the curve of rumen-reticular temperature relative to baseline (least squared means ± SE) by sampling time (at estrus or ovulation), THI and peak activity [**(A)**; *P* < 0.01], or duration [**(B)**; *P* < 0.01] obtained from a mixed linear regression model as described in Table 1. Superscripts of letters a-e denote significant differences (*P* < 0.05), while letters w-x denote tendencies (0.05 ≥ *P* < 0.10), between all groups of each panel. High peak activity: estrous expression greater than the median of 80 index on the AAM. Low peak activity: estrous expression less than the median. Long duration: estrous expression lasting longer than the median of 12 h. Short duration: estrous expression lasting less than the median. High THI: Maximum THI >72. Low THI: Maximum THI <72. THI data was collected relative to the sampling time (estrus vs. ovulation).

**Figure 6:**
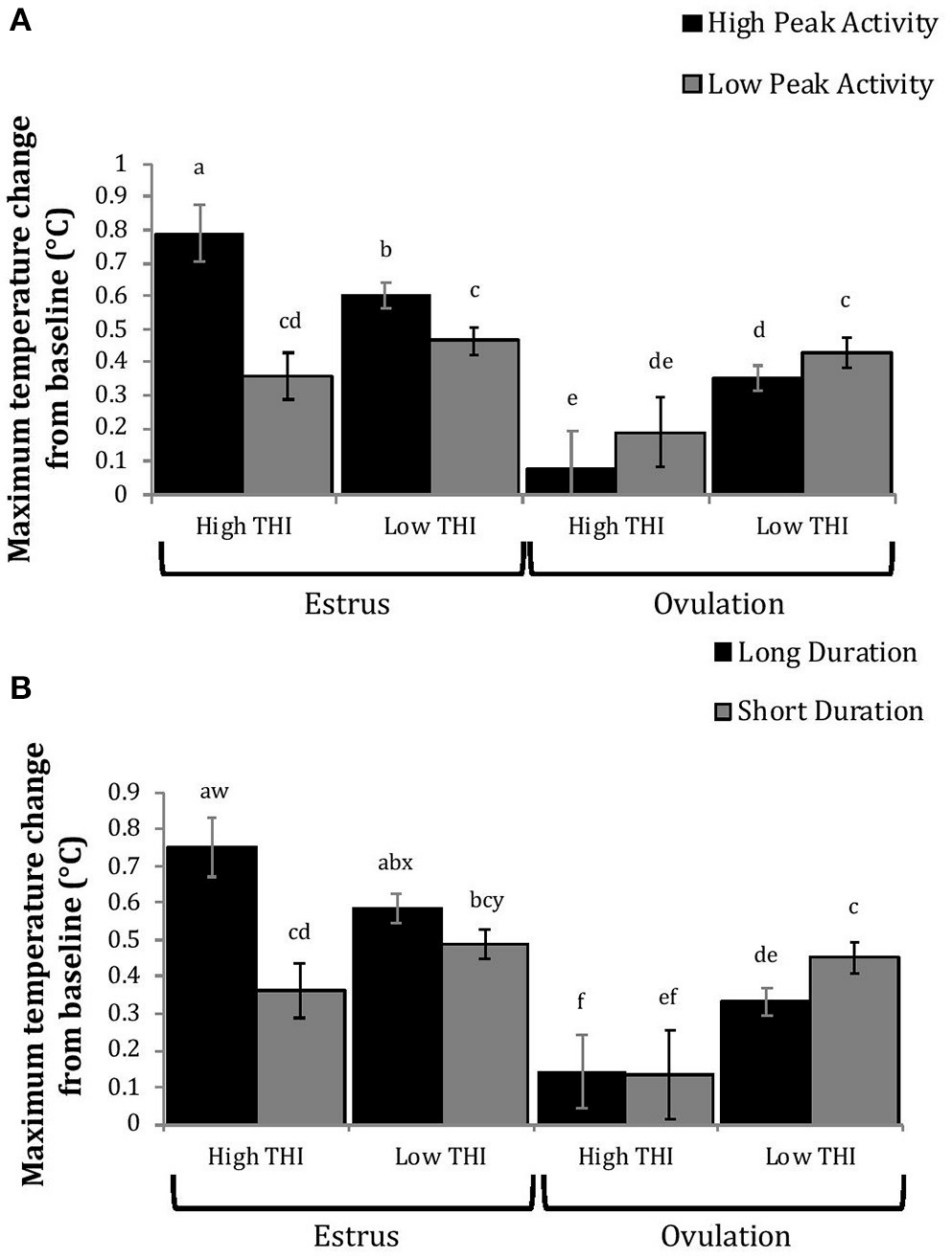
Maximum positive change in rumen-reticular temperature (least squared means ± SE) by sampling time (at the time of estrus or ovulation), THI and peak activity [**(A)**; *P* < 0.01], or duration [**(B)**; *P* < 0.01] obtained from a mixed linear regression model as described in Table 1. Superscripts of letters a-f denote significant differences (*P* < 0.05), while letters w-y denote tendencies (0.05 ≥ *P* < 0.10), between all groups of each panel. High peak activity: estrous expression greater than the median of 80 index on the AAM. Low peak activity: estrous expression less than the median. Long duration: estrous expression lasting longer than the median of 12 h. Short duration: estrous expression lasting less than the median. High THI: Maximum THI >72. Low THI: Maximum THI <72. THI data was collected relative to the sampling time (estrus vs. ovulation).

**Figure 7:**
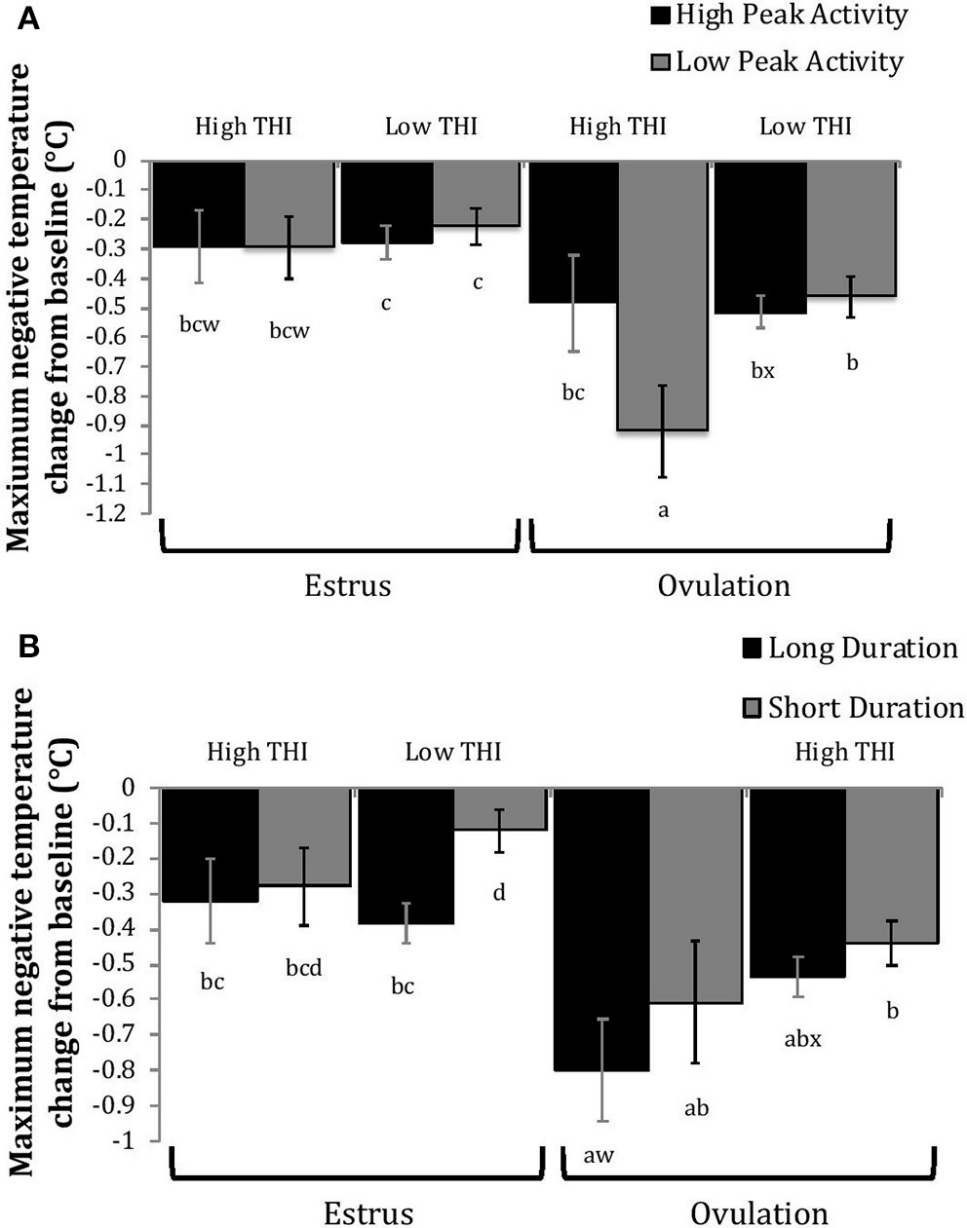
Maximum negative change in rumen-reticular temperature (least squared means ± SE) by sampling time (at the time of estrus or ovulation), THI and peak activity [**(A)**; *P* = 0.21], or duration [**(B)**; *P* = 0.37] obtained from a mixed linear regression model as described in Table 1. Superscripts of letters a-d denote significant differences (*P* < 0.05), while letters w-x denote tendencies (0.05 ≥ *P* < 0.10), between all groups of each panel. High peak activity: estrous expression greater than the median of 80 index on the AAM. Low peak activity: estrous expression less than the median. Long duration: estrous expression lasting longer than the median of 12 h. Short duration: estrous expression lasting less than the median. High THI: Maximum THI >72. Low THI: Maximum THI <72. THI data was collected relative to the sampling time (estrus vs. ovulation).

Cows with greater estrous expression, both intensity and duration, had greater AUC and PTC at the time of estrus than at ovulation, but cows with lesser estrous expression had similar AUC and PTC at estrus and ovulation (Figures 3, 4). Cows had more drastic declines in rumen-reticular temperature (NTC) at ovulation than at estrus, irrespective to estrous expression intensity or duration (Figures 3, 4). There was a three-way interaction present between sampling time, estrous expression and THI for AUC (Figure 5; *P* < 0.01) and PTC (Figure 6; *P* < 0.01), but not for NTC (Figure 7; *P* = 0.37); this was consistent whether estrous expression was measured using peak activity or duration of estrus.

Cows with greater estrous expression had greater AUC_E_ and PTC_E_ relative to those with lesser expression at the time of estrus, but the magnitude of these changes was associated with THI at estrus. On days with higher THI, cows with greater estrous expression had larger changes of both AUC_E_ and PTC_E_ than those with lesser estrous expression; on days with lower THI the effect of estrous expression on rumen-reticular temperature change was dampened (Figures 5, 6). Conversely, negative change in rumen-reticular temperature was small and not associated with either estrous expression or THI at the time of estrus; this may be due to a drop in temperature being associated with ovulation and not estrus.

Generally, when THI was considered low around the time of ovulation, positive changes in temperature (AUC_O_ and PTC_O_) tended to be lower for cows with greater estrous expression than those with lesser expression. Under high THI conditions, AUC_O_ and PTC_O_ were low and without difference between estrous expression intensities (Figures 5, 6). Negative temperature changes (NTC_O_) were more negative around ovulation than at estrus, but there were no consistent differences associated with estrous expression intensity or THI around ovulation (Figure 7). The associations of sampling time, estrous expression, and THI with AUC, PTC, and NTC have been depicted in Figures 5–7, respectively. Multiparous cows were found to have lower NTC than primiparous cows (-0.49 ± 0.05 vs. −0.36 ± 0.03 °C; *P* = 0.04) irrespective to sampling time; parity differences were not found for AUC or PTC. Similarly, milk production was found to be associated with PTC, where cows with higher milk yields had greater PTC; however, again there was no interaction found between this factor and sampling time. Stage of lactation, BCS, and GS all failed to impact AUC, PTC, or NTC and no interactions were found with sampling time.

### Temperature Alerts

**Figure 8:**
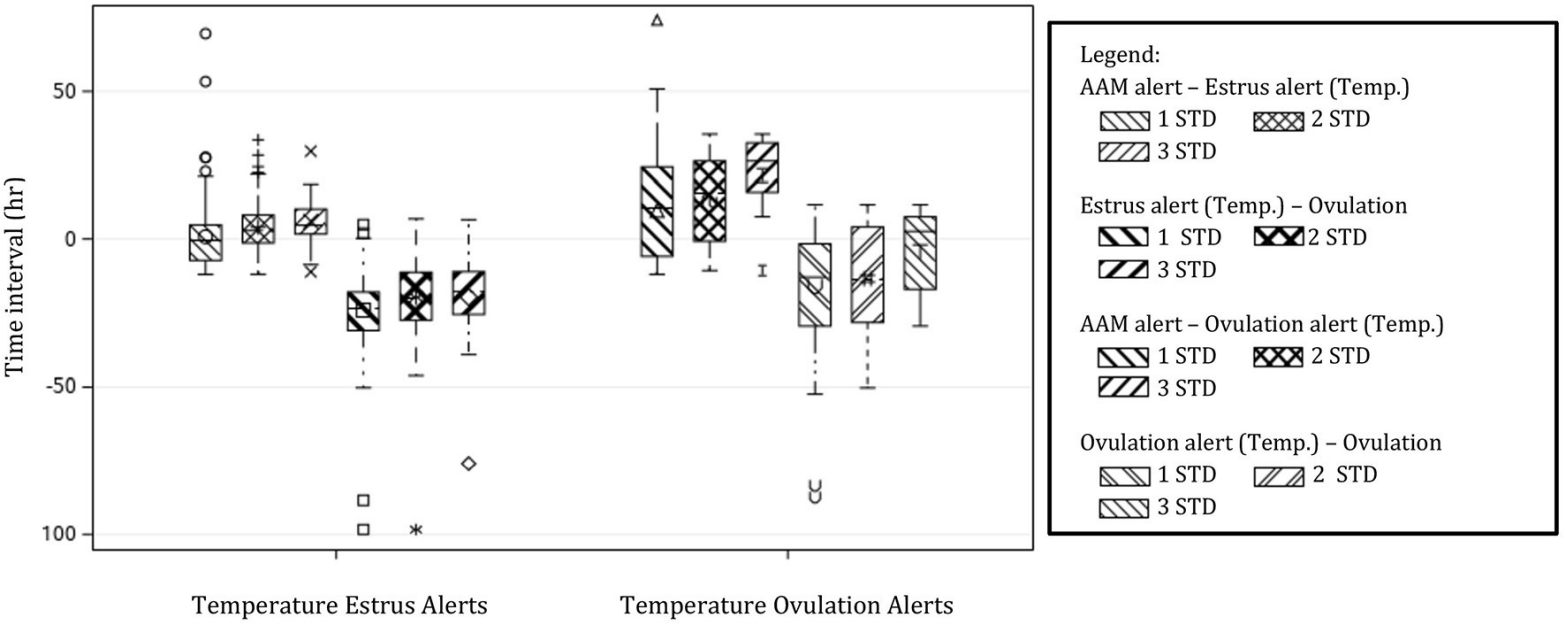
Boxplots demonstrating the distribution of time intervals between automated activity monitor (AAM) alerts, alerts for estrus, and ovulation based off of rumen-reticular temperature change, and the timing of ovulation. Temperature alerts were made from changes in rumen-reticular temperature for estrus (positive changes in temperature) and ovulation (negative changes) based on standard deviations (STD) from the baseline. Only thresholds of 1 STD, 2 STD, and 3 STD are depicted for simplicity.

Temperature alerts for estrus were found for 87.9, 76.8, 56.8, 35.3, 18.9, and 12.1% of estrus events when using 0.5 STD, 1 STD, 1.5 STD, 2 STD, 2.5 STD, and 3 STD from baseline as thresholds while temperature alerts for ovulation were found for 85.8, 65.2, 33.7, 18.9, 10.0, and 5.3% of events, respectively. Although more temperature alerts were found using the 0.5 STD threshold, alerts varied significantly. Even with the highest threshold for alert (3 STD), the precision of the estrus and ovulation alerts were not improved (i.e., smaller standard deviations from the mean were not found), and only a small proportion of events were alerted (Table 2). In general, temperature alerts for estrus occurred closer to AAM alerts but before the time of ovulation. Temperature alerts for ovulation occurred after AAM alerts and before ovulation. Temperature alerts for estrus varied less around the median in comparison with alerts for ovulation (Figure 8). The mean time (± SD) intervals between AAM alerts and the proportion of cows with alerts for estrus and ovulation based on changes in rumen-reticular temperature have been summarized in Table 2.

**Table 2:** Descriptive statistics of time intervals between the time of ovulation, automated activity monitor (AAM) alerts, and temperature alerts for estrus and ovulation based on standard deviation changes in rumen-reticular temperature in dairy cows.

**Interval**	**Alerted^**1**^ (%; *n*/*n*)**	**Mean time (h)**	**SD**	**Minimum**	**Maximum**
**AAM alert—temperature estrus alert**					
0.5 STD	87.9 (167/190)	−4.2	9.0	−12.0	45.3
1 STD	76.8 (146/190)	0.8	11.5	−12.0	69.5
1.5 STD	56.8 (108/190)	2.0	9.5	−12.0	29.2
2 STD	35.3 (67/190)	4.0	10.0	−11.9	33.6
2.5 STD	18.9 (36/190)	4.4	7.2	−11.9	21.3
3 STD	12.1 (23/190)	6.0	8.6	−11.1	29.8
**Temperature estrus alert—ovulation**					
0.5 STD	–	−29.3	12.8	−98.3	1.4
1 STD	–	−24.1	13.4	−98.3	4.9
1.5 STD	–	−22.5	14.1	−98.3	6.5
2 STD	–	−19.8	15.2	−98.3	6.8
2.5 STD	–	−20.5	13.8	−76.9	1.6
3 STD	–	−19.7	16.8	−75.9	6.5
**AAM alert—temperature ovulation alert**					
0.5 STD	85.8 (163/190)	2.7	14.0	−12.0	39.1
1 STD	65.2 (124/190)	9.7	16.9	−12.0	74.2
1.5 STD	33.7 (64/190)	10.4	16.9	−11.8	74.2
2 STD	18.9 (36/190)	13.0	15.1	−10.7	35.5
2.5 STD	10.0 (19/190)	18.1	15.0	−10.7	35.5
3 STD	5.3 (10/190)	21.6	14.2	−10.7	35.5
**Temperature ovulation alert—ovulation**					
0.5 STD	–	−23.1	18.1	−89.6	11.4
1 STD	–	−16.1	18.9	−87.6	11.6
1.5 STD	–	−17.1	20.9	−87.6	10.9
2 STD	–	−13.4	17.3	−50.2	11.6
2.5 STD	–	−9.4	15.3	−35.6	11.6
3 STD	–	−4.3	15.6	−29.5	11.6

*Alerts were determined on all data points between 12 h before the AAM estrus alert until 12 h after ovulation. On average, temperature estrus alerts occurred near the time of AAM alerts for estrus, but before ovulation, while temperature ovulation alerts occurred after AAM estrus alerts, and before ovulation*.

1 *Proportion of AAM estrus alerts which were also alerted based on changes in rumen-reticular temperature*.

## Discussion

This is the first study to report the impacts of intensity and duration of estrous expression measured using automated activity monitors on changes in body temperature during estrus and ovulation. The outcomes of this manuscript are supported by results of Suthar et al. (18) and Wrenn et al. (19) who found higher vaginal temperatures during estrus and lower vaginal temperature around ovulation in lactating dairy cows. Interestingly, Suthar et al. (18) carried out their study in a tie-stall barn, suggesting that changes in temperature around estrus may occur even in the absence of physical exertion. Previous research using vaginal temperature has accounted much of the increase in temperature at estrus to increased blood flow to the vagina caused by increased concentrations of estradiol (20); however, as the current study used rumen-reticular temperature, increased thermal conductance in the vagina is likely not the only factor impacting body temperature at the time of estrus. In agreement, Cooper-Prado et al. (21) and Randi et al. (22) also reported rumen-reticular and external auditory canal temperature to increase at the onset of estrus, respectively, further suggesting that thermal conductance is not the only reason for changes in temperature at estrus. Suthar et al. (18) also suggests that changes in temperature may not only be from physical activity but may be associated with factors that regulate gonadotropin-releasing hormone having an impact on central thermoregulation. Fisher et al. (23) demonstrated that the LH surge was associated with an increase in vaginal temperature, suggesting that LH may have thermogenic properties. However, in the current study, we did not find rumen-reticular temperature to be increased at estrus in cows with low estrous expression, suggesting that physical movement may be more important for changes in rumen-reticular temperature than for other measures of body temperature. The current study used AAM to detect estrus, thus, it is a possibility that cows which did not reach the alert threshold of the monitor were missed within our evaluations. However, we would expect that cows with such low activity changes would act similarly to those with low estrous expression, and thus have very minimal changes in rumen-reticular temperature during estrus.

Although not tested in this study, previous reports arising from our laboratory using similar AAM have demonstrated that greater estrous expression is associated with higher fertility (8, 24, 25) and lower anovulation rates (12, 26). Greater estrous expression in the current study was found to generate higher positive amplitude changes in temperature at estrus but have smaller increases in temperature around ovulation than cows with lesser estrous expression when under low THI conditions. Further research is warranted to establish if changes in temperature related to estrus and ovulation are associated with fertility. From the current study, we are not able to decipher if the heightened temperature response in animals with higher intensity and longer duration of estrous expression is solely due to increased movement or if there are other underlying factors.

Increased rumen-reticular temperature at estrus observed in the current study supports the results from previous research using vaginal temperature where temperature surges were associated with estrus and thus a predictor of ovulation in dairy cows. Fisher et al. (23) reported a high correlation between the time of LH surge and peak vaginal temperature in non-lactating dairy cows. The authors concluded that an increase in vaginal temperature occurred within 6 h of the LH surge. Using vaginal thermometers in heifers, Mosher et al. (5) reported that the interval between onset of temperature increase until ovulation was 21:01 h, whereas the interval from LH peak to the time of ovulation was 21:04 h. These results suggested that the onset of an increase in vaginal temperature may be a good proxy for the LH surge and thus ovulation. Additionally, (27) reported less variation in the interval from the LH surge to the onset of vaginal temperature spike compared with the interval from peak estradiol to the onset of vaginal temperature peak. In the present study, rumen-reticular temperature did increase at estrus and had characteristic decreases in temperature at ovulation, but no agreements between either positive or negative temperature alerts, using standard deviations from baseline, and ovulation times were found. Although a high proportion of estrus events captured by the AAM also displayed a positive (88%) or negative (85%) temperature alert, when using a lenient threshold, the time range between the temperature alerts and ovulation, at any threshold, was too wide to be used practically for breeding decisions or the detection of ovulation.

Similar to the current study, Talukder et al. (6) were able to obtain high sensitivity for estrus by measuring vulvar temperature using infrared technology, but unable to obtain acceptable values for specificity and the range in time from the estrus alert to ovulation was quite large (16–60 h), where 73% of ovulations occurred 24 to 47 h after the alert. Contrarily, Kyle et al. (28) reported by using a threshold change in vaginal temperature from baseline of 0.4°C for a minimum of 3 h they were able to obtain a detection sensitivity and a positive predictive value of 89.4 and 85.7%, respectively, which was higher sensitivity than using visual observations. Additionally, a recent study using changes in skin surface temperature demonstrated that they were able to increase their specificity and positive predictive values by including a necessary decrease in temperature within the prior 72 h (29). Although changes in temperature has potential for being an indicator of estrus, more research is necessary to decipher more accurate ways of automatically monitoring ovulation times in dairy cows. Future studies should include secondary parameters, such as activity, lying, rumination or feeding time, which could be used in unison with rumen-reticular temperature to make predictions more accurate. In addition, future research should be carried out on the associations of hormone profiles, such as estradiol and progesterone, with variations in body temperature at estrus and ovulation.

During days with greater THI, changes in rumen-reticular temperature at estrus were found to be greater than on days with lower THI. The impacts of estrous expression on these temperature changes were less drastic under cooler and less humid conditions. Contrary to this study, Cooper-Prado et al. (21) did not find an impact of ambient temperature on changes in rumen-reticular temperature at the time of estrus. Similarly, Sakatani et al. (30) did not find an impact of season on changes in vaginal temperature at estrus. However, they also did not find an impact of season on estrous expression, although non-lactating Japanese Black cows were used, thus it is possible that there are breed differences in terms of coping with changes in environmental temperatures. Under lower THI conditions, cows with high estrous expression had a smaller increase in rumen-reticular temperature at estrus than those in higher THI conditions. This may be attributed to animals being better able to dissipate heat in colder and less humid environments (31).

## Conclusions

This study demonstrated that positive changes in rumen-reticular temperature were higher at the time of estrus than around the time of ovulation while negative changes in rumen-reticular temperature were greater around ovulation than at estrus. Although positive changes in temperature at estrus was largely explained by the intensity of estrous expression, negative changes in temperature around ovulation were generally unaffected by the intensity of estrous expression. Estrus events characterized as having greater estrous expression had higher positive changes in temperature than those with low estrous expression; the magnitude of these temperature changes was impacted by the environmental temperature and humidity on the day of estrus. Although differences in rumen-reticular temperature can be found at estrus and around ovulation, the wide variation in the timing of alerts based of temperature until ovulation prevents us from recommending this technique as an early alerting system for estrus or ovulation at this time. Future research should focus on how rumen-reticular temperature can be used more efficiently and accurately in determining the timing of ovulation, as well as a more in depth analyses using temperature in parallel with other automated sensors.

## Data Availability Statement

The raw data supporting the conclusions of this article will be made available by the authors, without undue reservation.

## Ethics Statement

The animal study was reviewed and approved by University of British Columbia's Animal Care Committee.

## Author Contributions

TB was responsible for experimental design, data collection, analysis, and writing of the manuscript. MK and LP were responsible for data collection and manuscript editing. RC was the lead supervisor responsible for experimental design and conceptualization of ideas and analyses and editing of the manuscript. All authors read and approved the final manuscript. All authors contributed to the article and approved the submitted version.

## Conflict of Interest

The authors declare that the research was conducted in the absence of any commercial or financial relationships that could be construed as a potential conflict of interest.
